# Alcohol use and mental health among older American adults during the early months of the COVID-19 pandemic

**DOI:** 10.1093/geroni/igaa057.3422

**Published:** 2020-12-16

**Authors:** Marisa Eastman, Lindsay Kobayashi, Jessica Finlay

**Affiliations:** University of Michigan, Ann Arbor, Michigan, United States

## Abstract

We investigated the association of self-reported changes in alcohol consumption with the prevalence of anxiety, depression, and loneliness in the early months of the COVID-19 pandemic among middle-aged and older US adults. Between April and May 2020, 6,938 US adults aged 55+ completed online questionnaires in the COVID-19 Coping Study, a national cohort study of older adults’ mental health and well-being. Multinomial logistic regression estimated self-reported changes in the frequency of alcohol consumption relative to before the pandemic, according to anxiety (5-item Beck Anxiety Inventory), depression (8-item Center for Epidemiologic Studies Depression Scale), and loneliness (3-item UCLA Loneliness Scale). All models were population-weighted and adjusted for confounders. Nearly half (46%) of adults reported drinking 1-7 drinks/week prior to the pandemic, 12% reported drinking 8+ drinks/week, and 42% reported not drinking. One in five adults (21%) reported a change in their alcohol consumption since the start of the pandemic, while 38% indicated they were drinking the same amount, and 42% reported not drinking alcohol. Older adults who screened positive for each of anxiety, depression, and loneliness reported drinking more than usual (OR=1.92; 95% CI: 1.92–1.93 for anxiety; OR=2.67; 95% CI: 2.67–2.68 for depression; OR=2.46; 95% CI: 2.45–2.46 for loneliness), compared to drinking the same as before the pandemic. These results demonstrate potentially negative changes in alcohol intake among middle-aged and older adults experiencing mental health symptomology during the early months of the COVID-19 pandemic.

